# Insight into the Role of the STriatal-Enriched Protein Tyrosine Phosphatase (STEP) in A_2A_ Receptor-Mediated Effects in the Central Nervous System

**DOI:** 10.3389/fphar.2021.647742

**Published:** 2021-04-19

**Authors:** Maria Rosaria Domenici, Cinzia Mallozzi, Rita Pepponi, Ida Casella, Valentina Chiodi, Antonella Ferrante, Patrizia Popoli

**Affiliations:** ^1^ National Centre for Drug Research and Evaluation, Istituto Superiore di Sanitá, Rome, Italy; ^2^ Department of Neuroscience, Istituto Superiore di Sanità, Rome, Italy

**Keywords:** adenosine A_2A_ receptor, striatal-enriched protein tyrosine phosphatase, brain, SH-SY5Y neuroblastoma cell lines, functional interaction

## Abstract

The STriatal-Enriched protein tyrosine phosphatase STEP is a brain-specific tyrosine phosphatase that plays a pivotal role in the mechanisms of learning and memory, and it has been demonstrated to be involved in several neuropsychiatric diseases. Recently, we found a functional interaction between STEP and adenosine A_2A_ receptor (A_2A_R), a subtype of the adenosine receptor family widely expressed in the central nervous system, where it regulates motor behavior and cognition, and plays a role in cell survival and neurodegeneration. Specifically, we demonstrated the involvement of STEP in A_2A_R-mediated cocaine effects in the striatum and, more recently, we found that in the rat striatum and hippocampus, as well as in a neuroblastoma cell line, the overexpression of the A_2A_R, or its stimulation, results in an increase in STEP activity. In the present article we will discuss the functional implication of this interaction, trying to examine the possible mechanisms involved in this relation between STEP and A_2A_Rs.

## Introduction

P1 adenosine receptors are the most investigated purinergic receptors within the central nervous system (CNS). Since their identification in the late 70s, they have been the subject of numerous studies that established their widespread distribution in the brain and their pivotal role in the functioning of the CNS. The adenosine A_2A_ receptor (A_2A_R) is one of the four G protein coupled receptor subtypes (A_1_, A_2A_, A_2B_, and A_3_), it is coupled with G_s_ protein and its stimulation activates adenylate cyclase causing an increase in intracellular cAMP levels (Borea et al., 2018). With the exception of the dorsal and ventral striatum, where A_2A_R is present at remarkably high levels, in the rest of the brain the expression of the receptor is quite low (Rosin et al., 2003). Despite this, the huge importance of A_2A_R in the CNS is witnessed by its role in the regulation of fundamental functions such as movement, cognition and emotions and, for this reason, it has attracted the interest of researchers as a potential therapeutic target (Borah et al., 2019). Indeed, the A_2A_R antagonist istradefylline (Nourianz®) has recently been approved in the United States, after its first registration in Japan, for the treatment of Parkinson’s disease, as an add-on to levodopa (Chen and Cunha, 2020).

One of the peculiarities of A_2A_R is the ability to modulate the activation and function of several other receptors, such as dopamine D2, cannabinoid CB1, metabotropic glutamate 5 receptor (mGlu_5_R), as well as adenosine A_1_ receptors, by forming heteroreceptor complexes (Cabello et al., 2009; Tebano et al., 2012; Moreno et al., 2018; Ferré and Ciruela, 2019). Recently, we identified a novel role of A_2A_Rs in the rodent brain and in neuronal cells. Specifically, we demonstrated that the stimulation of A_2A_Rs results in the activation of the STriatal-Enriched protein tyrosine phosphatase STEP, a brain-specific tyrosine phosphatase involved in several functions, including learning and memory (Goebel-Goody et al., 2012; Chiodi et al., 2014; Mallozzi et al., 2020).

In this article we will present some recent results on the A_2A_Rs/STEP interaction and on the possible mechanisms involved. The physiological implication of this new receptor function will be discussed.

## STriatal-Enriched Protein Tyrosine Phosphatase

**FIGURE 1:**
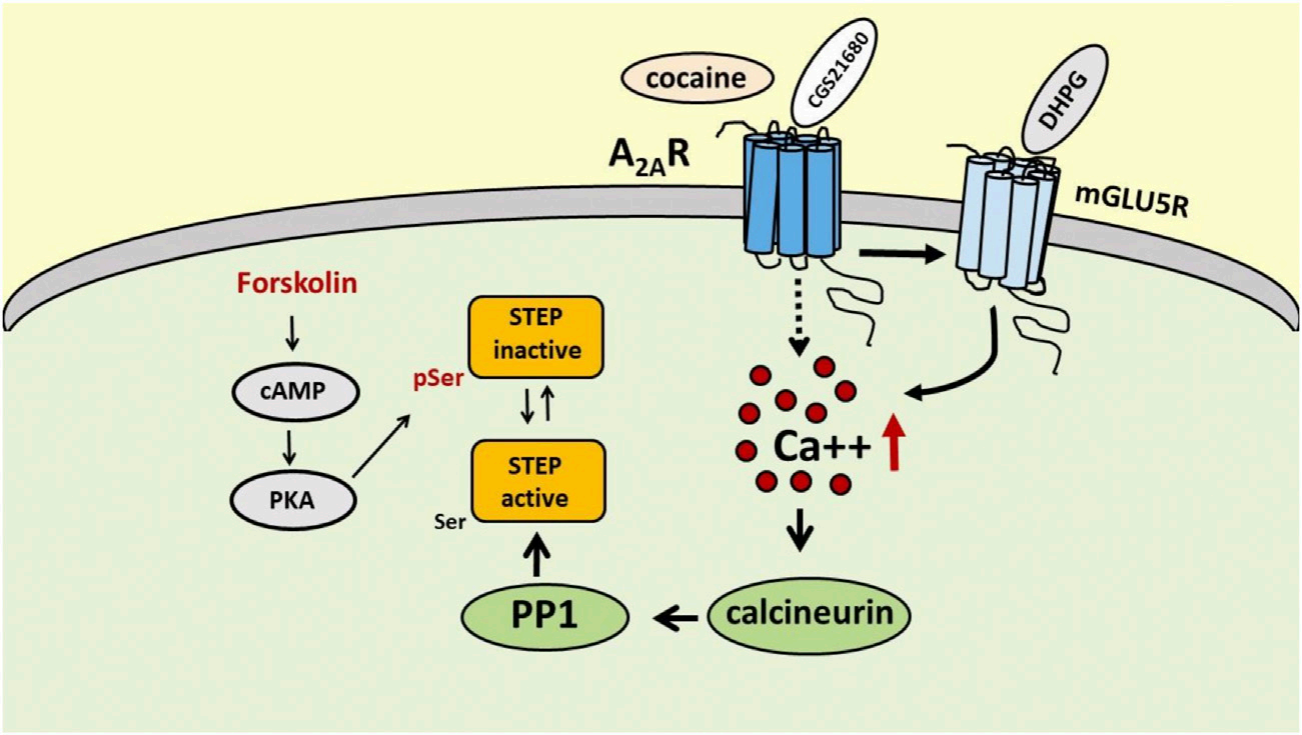
Schematic representation of the possible mechanisms involved in the regulation of STEP activity by A_2A_Rs. Activation of A_2A_Rs, directly with the selective A_2A_R agonist CGS 21680 or indirectly with cocaine, increases STEP activity through a peculiar mechanism involving mGlu5R, intracellular Ca^++^ increase and calcineurin recruitment. On the contrary, activation of PKA by forkolin promotes STEP inactivation through phosphorylation of Ser residue.

In the early 90s, Paul J. Lombroso and collaborators isolated a new protein tyrosine phosphatase in the brain, particularly enriched in the striatum, that strongly colocalized with DARPP32 and tyrosine hydroxylase-positive neurons, which was denominated STEP (Lombroso et al., 1991, 1993). STEP exists in several isoforms that differ in intracellular localization and functions, and all originate by alternative splicing of a single *Ptpn5* gene (Boulanger et al., 1995). The two major isoforms are STEP61, associated with membrane compartments, and the cytosolic protein STEP46, and both carry the consensus sequence required for the phosphatase catalytic activity and a kinase-interacting motif (KIM), that allows the interaction with the substrates. When phosphorylated at the specific Ser residues (221 for STEP61 and 49 for STEP46) within the KIM domains, STEP61 and STEP46 become inactive since they lose their ability to bind to the substrates (Bult et al., 1996; Pulido et al., 1998; Kamceva et al., 2016). STEP activity is regulated by quite complex phosphorylation/dephosphorylation mechanisms, in which calcineurin (a calcium/calmodulin-activated serine/threonine phosphatase, also known as PP2B) and protein kinase A (PKA) play a major role (Figure 1). Calcineurin activates STEP through protein phosphatase 1 (PP1), which dephosphorylates the regulatory serine residue and activates STEP (Paul et al., 2000). The activation of PKA results in the inhibition of STEP activity either through the direct phosphorylation of STEP61 and STEP46 at the specific serine residues and, indirectly, through the phosphorylation of DARPP-32 and the inhibition of PP1(Paul et al., 2000; Valjent et al., 2005; Giralt et al., 2011). Several neurotransmitter receptors, such as dopamine D1 receptor and nicotinic α7 nAChR, are able to modulate STEP activity (Paul et al., 2000; Zhang et al., 2013). Moreover, mGlu_5_R has been shown to increase STEP translation at dendritic levels that mediates AMPA receptor endocytosis, a mechanism that could be involved in DHPG-induced LTD. (Moult et al., 2002; Zhang et al., 2008; Goebel-Goody et al., 2012; Chen et al., 2013). As already mentioned, and as we will discuss later, STEP activity is also modulated by A_2A_R (Chiodi et al., 2014; Mallozzi et al., 2020).

Several substrates of STEP have been identified. The glutamate receptor subunits GluN2B and GluA1/GluA2 of the NMDA and AMPA receptors, respectively, are important STEP substrates whose dephosphorylation at specific tyrosine residues promotes receptor internalization and reduces NMDA- and AMPA-mediated synaptic transmission, having a strong impact on synaptic plasticity (Won and Roche, 2021). Pyk2 and Fyn, two other STEP substrates, are also involved in the modulation of synaptic transmission and synaptic plasticity by influencing several mechanisms, including the direct or indirect phosphorylation of glutamate receptor subunits (Huang et al., 2001; Giralt et al., 2017; Matrone et al., 2020). Moreover, STEP shows a role at presynaptic level, modulating glutamate neurotransmitter release (Bosco et al., 2018). Additional STEP substrates are the extracellular signal-regulated kinases 1 and 2 (ERK1/2), involved in memory processes and in synaptic plasticity, and p38, implicated in cell death and survival, and both are inactivated by dephosphorylation of specific tyrosine residues upon STEP activation (Goebel-Goody et al., 2012).

STEP appears to be strongly involved in neurological disorders where synaptic dysfunctions have been identified, as well as in diseases where excitotoxicity play a major role.

Indeed, in the last years a dysregulation of STEP has been found in several neuropsychiatric diseases and its modulation, by genetic or pharmacological tools, was accompanied by the attenuation of the symptoms in animal models of diseases (Karasawa and Lombroso, 2014; Kulikova and Kulikov, 2017). The general idea is that elevated STEP levels or activity have detrimental effects on cognition by negatively influencing synaptic strengthening through the dephosphorylation of substrates regulating synaptic plasticity (Pelkey et al., 2002; Fitzpatrick and Lombroso, 2011). Indeed, high levels of STEP expression have been found in animal models of Alzheimer’s and Parkinson’s diseases (neurodegenerative diseases characterized by cognitive impairment), and in the hippocampus of aged mice, rats and rhesus monkeys and in the brain of individuals with mild cognitive impairment (Xu et al., 2012; Kurup et al., 2015; Castonguay et al., 2018). Furthermore, STEP over-expression induced memory deficits in mice, and its inhibition ameliorates memory performances in aged rats and in animal models of neuropsychiatric diseases (Castonguay et al., 2018). However, during aging reduced STEP activity and expression have also been reported (Rajagopal et al., 2016; Cases et al., 2018).

Beside its role in modulating synaptic plasticity and cognition, STEP is emerging as a key regulator of neuronal survival and death. As demonstrated by Choi et al. (2007), STEP increases neuronal vulnerability to excitotoxic cell death in primary hippocampal cultures and the sensitivity of neurons to excitotoxicity induced by Status Epilepticus in mice. These effects were due to the blockade of neuroprotective responses initiated by the ERK/MAPK signaling pathway. On the other hand, in an *in vivo* model of cerebral ischemia, where excitotoxic cell death plays a major role, STEP exerts a neuroprotective effect by inhibiting the p38 MAPK signaling pathway. In fact, administration of the STEP-derived peptide prevents p38 MAPK activation and reduces ischemic brain damage in STEP KO mice (Deb et al., 2013). In order to reconcile those apparently conflicting results, it should be considered that, depending on the level of calcium increase following NMDA receptors stimulation, STEP activity can be increased and promote neuroprotection by reducing p38 activation or, in case of a prolonged insult, the resulting STEP degradation will facilitate cell death pathways by increasing the phosphorylation of p38 MAPK (Poddar et al., 2010). In addition, the stimulation of synaptic or extrasynaptic NMDA receptors differently impacts on STEP expression, resulting in the activation of ERK1/2 or p38 MAPK, respectively, and promoting cell survival or death (Xu et al., 2009).

Another well identified role for STEP is the modulation of the effects of psychostimulant drugs such as cocaine and amphetamine (Valjent et al., 2005; Hopf and Bonci, 2009; Sun et al., 2013; Siemsen et al., 2018). As for cocaine effects, initial studies demonstrated that following acute cocaine treatment in mice, the increase in ERK1/2 phosphorylation (pERK1/2) in a subpopulation of dopamine D1R-containing striatal neurons was mediated, at least in part, by D1R-mediated STEP inactivation (Valjent et al., 2005). However, in condition of chronic cocaine consumption, such as in models of cocaine self-administration, a decrease in STEP phosphorylation and pERK1/2 are observed in the rat prefrontal cortex, that could represent early events in withdrawal mechanisms (Sun et al., 2013). More recently, cocaine-induced STEP activation has been demonstrated in the early phase of abstinence, which mediates the decrease in p-ERK observed in the pre-limbic cortex of cocaine-seeking rats (Siemsen et al., 2018). These studies demonstrate an active role of STEP in cocaine-mediated effects. In line with this, as we will describe below, we found that the synaptic depression exerted by cocaine in the striatum involved STEP activation through the stimulation of A_2A_Rs (Chiodi et al., 2014), suggesting an interaction between the receptor and the phosphatase.

## Evidence of a Functional Interaction Between A_2A_R and STriatal-Enriched Protein Tyrosine Phosphatase

The first evidence of an involvement of STEP in A_2A_R-mediated effects came from our study investigating the synaptic effects of cocaine in the striatum (Chiodi et al., 2014). We found that cocaine reduced striatal synaptic transmission, evaluated by recording extracellular field potentials and AMPA- and NMDA-mediated currents in whole cell patch-clamp experiments in corticostriatal slices. Cocaine effects were reduced by A_2A_R antagonist, by inhibitor of protein tyrosine phosphatases, by a calcineurin inhibitor and by TAT-STEP, a substrate trapping mutant peptide that makes STEP enzymatically inactive. In addition, the effect of cocaine was strongly reduced in A_2A_R knock-out mice. In order to understand the relationship among cocaine, A_2A_Rs and tyrosine phosphatases (and STEP in particular), we evaluated the enzimatic activity of the total tyrosine phosphatases, and of STEP in particular, in mice striatal tissue after cocaine stimulation. We could show that cocaine increased tyrosine phosphatase activity, and in particular STEP activity, in A_2A_R-dependent manner. In fact, cocaine failed to activate STEP in the presence of the A_2A_R antagonist or in A_2A_R knock-out mice. These results suggested that a possible mechanism through which cocaine reduced synaptic transmission is the recruitment of A_2A_R and STEP activation. Indeed, STEP activation results in the dephosphorylation and internalization of NMDA and AMPA receptor subunits causing depression of excitatory synaptic transmission (Zhang et al., 2008; Zhang et al., 2010; Zhang et al., 2011; Kurup et al., 2010). Moreover, the involvement of calcineurin suggests the need of intracellular calcium increase. These mechanisms have been very nicely examined and depicted by Robert Yasuda (Yasuda, 2020) who recapitulated the way through which A_2A_R modulates cocaine-induced synaptic depression and, possibly, cocaine self-administration, via STEP activation.

In a recent paper, in order to confirm and further investigate the relationship between A_2A_R and STEP, we used cellular, genetic, and pharmacological approaches to evaluated STEP activity in different condition of A_2A_R stimulation and in different brain areas (Mallozzi et al., 2020). We took advantage of a transgenic rat strain overexpressing A_2A_R in the brain (Chiodi et al., 2016) in which we evaluated STEP activity in the striatum and hippocampus. In basal conditions, we found a significant increase in STEP activity in the striatum and hippocampus of A_2A_R overexpressing rats with respect to wild type. Moreover, in the striatum the selective A_2A_R agonist CGS21680 increased STEP activity in wild type but not in A_2A_R overexpressing rats (where STEP activity was already high), while ZM241385, the A_2A_R antagonist, reduced STEP activity in overexpressing rats (up to wild type levels), without any effects in wild type animals. In addition, in A_2A_R overexpressing rats we found a decrease in the phosphorylation levels of GluN2B and Pyk2, two well-known STEP substrates, consistent with an increased phosphatase activity (Mallozzi et al., 2020).

Similar results have been obtained in the neuroblastoma cell line SH-SY5Y, which expresses both STEP and A_2A_Rs, where we confirmed that the stimulation of A_2A_R with CGS21680 causes an increase in STEP activity, evaluated also by western blotting analysis as a decrease in STEP phosphorylation status.

An interesting point to address is by which mechanism the stimulation of A_2A_Rs results in STEP activation. It is demonstrated, in fact, that the activation of the cAMP/PKA pathway, as it occurs with the activation of G_s_-coupled receptors (and the A_2A_R belongs indeed to the family of G_s_-coupled receptors), rather results in the phosphorylation and inactivation of STEP (Paul et al., 2000). Actually, also in our hands the treatment of SH-SY5Y cells with forskolin (Mallozzi et al., 2020), which induces activation of the cAMP/PKA pathway, causes an up-regulation of phosphoSTEP, consistent with the inactivation of the phosphatase. Thus, a different mechanism must be hypothesized to explain A_2A_R-mediated STEP activation.

To assess if a physical interaction between A_2A_R and STEP could be necessary, we performed Bioluminescence Resonance Energy Transfer (BRET) assays (Molinari et al., 2008; Casella et al., 2011) in SH-SY5Y cell populations co-expressing a green fluorescent version of STEP61 with either luminescent-A_2A_R (a kind gift from Francisco Ciruela) or luminescent-β-arrestin 2 protein (a well recognized G-protein independent signal transducer) (Sachs et al., 2005). In our experiments, exposure of these cells to the A_2A_R agonist CGS21680 failed to enhance the BRET signal over the level of unstimulated samples, suggesting that STEP61 is probably not an A_2A_R interacting partner (unpublished data) and that the signaling route of A2AR to STEP61 probably does not depend on their direct interaction. However, to definitively exclude a direct interaction between A_2A_R and STEP, BRET experiments should be performed also by using other STEP isoforms (i.e., STEP46).

In a recent paper Won and collaborators used mass spectrometry to study STEP binding proteins and identified 315 candidate proteins and, among them, the authors recognized mGlu_5_R as an interactor of STEP (Won et al., 2019). This finding is particularly interesting since it is well known that A_2A_R and mGlu_5_R physically and functionally interact in several brain areas, that activation of A_2A_Rs exerts a permissive role on mGluR_5_R-mediated effects (Ferre et al., 2002; Domenici et al., 2004; Tebano et al., 2005; Krania et al., 2018) and, most importantly, that mGlu_5_R stimulation results in an increase in STEP translation and, presumably, activation (Zhang et al., 2008). Moreover, mGlu_5_R interacts with Gq proteins and its stimulation enables the activation of PLC signaling and intracellular calcium increase (Conn and Pin, 1997). Interestingly, in our recent paper we found that A_2A_R-induced STEP activation is calcium-dependent since in SH-SY5Y cells it is prevented by the calcium chelator BAPTA-AM and by the calcineurin inhibitor FK506 (Mallozzi et al., 2020). Thus, on the basis of this calcium dependence, the mGlu_5_R could be a good candidate to mediate A_2A_R effects on STEP activity. Therefore, in preliminary experiments we verified in the SH-SY5Y cell line the effect of the selective A_2A_R agonist CGS 21680 on STEP activity in the presence of the mGlu_5_R antagonist MPEP, and we found that by blocking mGlu_5_R, CGS 21680 was no longer able to increase STEP activity (unpublished results). Even though additional experiments are needed, these results clearly suggest that A_2A_Rs modulate STEP activity through the involvement of mGlu_5_R (Figure 1).

## Discussion and Conclusion

The studies presented above provide a clear demonstration of a functional interaction between A_2A_Rs and STEP in the striatum and hippocampus of the rat and mouse brain, which has been confirmed in the SH-SY5Y neuroblastoma cell line, suggesting that this interaction can occur in different cell types. The mechanism through which A_2A_R and STEP interact is still not clearly identified, but the calcium dependence and the involvement of mGlu_5_R are both very likely. Even though a strong evidence that this interaction occurs also *in vivo* is still lacking, a review of the scientific literature shows that in some neuropathologic conditions STEP and the A_2A_R are dysregulated in a similar way. For example, STEP levels are elevated in rodent models of Alzheimer’s disease, in postmortem brains of patients with Alzheimer’s disease and in the brain of individuals with mild cognitive impairment (Zhang et al., 2011; Xu et al., 2012; Castonguay et al., 2018). In the same way, A_2A_Rs are upregulated in Alzheimer’s disease, both in animal models and in the brain of patients (Arendash et al., 2006; Albasanz et al., 2008; Orr et al., 2015; Temido-Ferreira et al., 2020). More interestingly, during aging both STEP and A_2A_Rs are upregulated and show an enhanced activity in animal models and in the human aged brain, and inhibition of STEP activity or the blockade of A_2A_Rs improved memory performances (Castonguay et al., 2018; Orr et al., 2018; Ferré and Ciruela, 2019; Temido-Ferreira et al., 2019; Temido-Ferreira et al., 2020). Finally, STEP over-expression induced memory impairment in adult mice (Castonguay et al., 2018), and the same occurs in conditions of increased A_2A_Rs activation (Gimenez-Llort et al., 2007; Li et al., 2015; Pagnussat et al., 2015). Accordingly, in A_2A_R overexpressing rats, in which we demonstrated an increased basal STEP activity in the striatum and hippocampus, working memory deficits have been reported (Gimenez-Llort et al., 2007; Mallozzi et al., 2020). Very recently, Ferrante et al. (2021) demonstrated that STEP protein expression and activity were increased in Fragile X mice and normalized by the A_2A_R antagonist KW6002 treatment, which improved the behavioral phenotype as well.

Thus, one important conclusion is that the modulation of STEP activity could contribute to the effects of A_2A_Rs on cognitive functions (Chen, 2014; Uchida et al., 2014; Temido-Ferreira et al., 2019). As for Parkinson’s disease, an interesting consideration is that long-term treatment of patients with istradefylline could result not only in the improvement of motor deficits but also in beneficial effects on cognitive dysfunction, and that the inhibition of STEP could play a major role in this effect. In fact, STEP levels are increased in human brains and in animal models of Parkinson’s disease, which may contribute to the cognitive impairment that occurs in the disease (Kurup et al., 2015).

In conclusion, the interaction between A_2A_R and STEP (possibly through the involvement of mGlu_5_R) could have clinical relevance and its possible consequences should be contemplated when proposing drugs targeting the A_2A_Rs. Notably, particular attention should be payed when considering A_2A_R agonists as potential treatment for human pathologies (Borea et al., 2018; Borah et al., 2019), given their potential to impair cognitive performance by increasing STEP activity.

## Data Availability

The original contributions presented in the study are included in the article/Supplementary Material, further inquiries can be directed to the corresponding author.
